# Assessing the Risk and Awareness of Type 2 Diabetes Mellitus Among Medical Students in Riyadh, Saudi Arabia

**DOI:** 10.7759/cureus.39087

**Published:** 2023-05-16

**Authors:** Abdulrahman S Algadheeb, Karam M Basham, Mohsin A Alshahrani, Ahmed A Alshamrani, Ahmed Alzahrani, Sultan S Algadheeb, Mohammad A AlRefaei

**Affiliations:** 1 Family Medicine, Prince Sultan Military Medical City, Riyadh, SAU; 2 Family Medicine, King Saud Medical City, Riyadh, SAU; 3 Family Medicine, Ministry of Health, Third Cluster, Riyadh, SAU; 4 College of Medicine, Alfaisal University, Riyadh, SAU

**Keywords:** type 2 diabetes, saudi arabia, risk assessment, medical student, awareness

## Abstract

Background

Metabolic diseases, such as diabetes mellitus (DM), are increasingly becoming a global problem. Apart from clinical judgment, it is necessary to have reliable, inexpensive, and non-invasive tools to assess the risk of type 2 diabetes mellitus (T2DM), as the disease can be diagnosed years after its onset, with irreversible complications.

Methodology

This is an observational cross-sectional study conducted at the College of Medicine, King Saud University, located in the capital city of Saudi Arabia. Data was collected through a questionnaire administered to medical students who voluntarily agreed to participate. The risk of T2DM was assessed using the American Diabetes Association diabetes risk test. The collected data was coded, entered into the Statistical Package for Social Sciences software (SPSS; IBM Inc., Armonk, New York), and subsequently analyzed.

Results

A total of 417 participants were included in our study, with a mean age of 20 ± 2.03 years and a mean body mass index (BMI) of 24.2 ± 5.3. The mean DM risk score was 1.83 ± 1.32 out of a total of 11. Of all the participants, 98.8% had a low-risk score for T2DM, while only 1.2% were identified as having a higher risk of developing T2DM. Approximately 77% of the participants had checked their weight and calculated their BMI in the last year. Among the participants, 98.1% identified obesity as a risk factor for T2DM, 57.8% reported smoking as a risk factor, 96.4% recognized a family history of DM as a risk factor, 80.8% identified a history of gestational DM as a risk factor, and 53.7% reported hypertension as a risk factor for T2DM.

Conclusions

Most of the study participants demonstrated a good knowledge level and awareness regarding T2DM, with only 1.2% found to be at an increased risk of developing the disease. Our analysis did not identify any significant association between having a high or low-risk score for T2DM and having a high or low awareness level of the disease.

## Introduction

Diabetes mellitus (DM) is a chronic metabolic disorder characterized by high blood sugar levels and impaired metabolism of carbohydrates, lipids, and proteins due to insufficient insulin secretion and/or insulin action. The disease has two primary forms: type 1 diabetes mellitus (T1DM) and type 2 diabetes mellitus (T2DM). T2DM is the most prevalent form, accounting for 90-95% of all cases of diabetes [1-2]. Numerous studies have predicted a rising prevalence of DM, particularly in developing countries [3]. It is estimated that between 2010 and 2030, developed countries will experience a 20% increase in the number of adults with diabetes, while developing countries will experience a much larger 69% rise [4]. The projected number and proportion of adults with diagnosed diabetes in the United States are expected to increase from 22.3 million (9.1%) in 2014 to 39.7 million (13.9%) by 2030 and to 60.6 million (17.9%) by 2060 [5]. The Middle East and North Africa region has the highest prevalence of diabetes in the world, as well as the highest adjusted mortality rate from non-communicable diseases. It also has the second-highest rate of increase in diabetes prevalence globally [6]. As the prevalence of diabetes is expected to rise in the future, it is imperative to evaluate the younger generation's risk factors and knowledge of the disease to raise awareness and encourage them to take preventive measures, ultimately reducing the incidence of the disease in the future.

A study conducted at the University of Ajman, United Arab Emirates, enrolled 182 students, of which only 55% were found to be aware that obesity is a risk factor for DM, and only 53% were aware that decreased physical activity is also a risk factor for DM [7]. A survey was carried out in Muscat, Oman, to evaluate the understanding of type 2 diabetes among high school students using a questionnaire with a scoring system. The results indicated that only 24% of the students achieved a score of more than 10 out of 20, despite 82% of them admitting to having limited knowledge of diabetes [8]. A cross-sectional study was conducted in Damascus, Syria, involving 275 medical students to assess their knowledge of diabetes diagnosis. The findings revealed that a significant proportion of the students lacked knowledge in this area. Only 37.8% correctly identified that a fasting plasma glucose level of 126 mg/dl or above is diagnostic for diabetes. Similarly, only 36.7% correctly identified that an HbA1c level of 6.4% or above indicates diabetes and only 34.2% correctly identified that an oral glucose challenge after two hours or a random plasma glucose level of 200 mg/dl or above is diagnostic for diabetes [9]. Though the risk of developing DM is higher in the older population, the young population is still at risk, which could be attributed to non-healthy lifestyles on a background of genetic predisposition. The level of education and awareness has also been considered an important modifiable factor for developing T2DM with an inverse relationship. Therefore, we conducted this study to assess T2DM risk and the level of awareness among young Saudi medical students in Riyadh, Saudi Arabia.

## Materials and methods

Study design and selection criteria

The study was conducted as a cross-sectional study at the College of Medicine, King Saud University in Riyadh, Saudi Arabia. Its objective was to assess the awareness level of risk factors for T2DM among medical students and to determine the relationship between having a high- or low-risk score for T2DM and having a high or low awareness level of the disease. The study inclusion criteria comprised medical students who had agreed to participate. Non-medical students and those who refused to participate were excluded.

Study population and sample size 

The population consisted of participants who fulfilled the inclusion and exclusion criteria. The sample size was determined using statistical software for the epidemiology info program, based on a 95% confidence interval, a 5% margin of error, and the total selected population. The estimated sample size was 384, which was adjusted to 422 to account for a 10% non-response rate. This study design and sample size provided a robust representation of the target population and enabled meaningful statistical analyses of the prevalence and determinants of the health condition of interest.

Questionnaire 

The primary data collection tool utilized in this study was a self-administered questionnaire. The questionnaire was validated based on previous studies and included questions about the socio-demographic characteristics of the participants, such as age group, gender, nationality, and residence, as well as their level of awareness of T2DM risk factors. To assess the risk of T2DM, we used the American Diabetic Association diabetes risk test [10]. Prior to administering the questionnaire, a pilot study was conducted with a sample of 20 participants whose results were not included in the final analysis. Any necessary modifications were made to ensure the clarity and comprehensibility of the questions.

Data management and statistical analysis

The data was collected using a convenient non-probability sampling technique. After collection, the data was coded, entered, and analyzed using the Statistical Package for Social Science (SPSS) version 23 (IBM Inc., Armonk, New York). Qualitative data was presented in the form of numbers and percentages, and the Chi-squared (χ2) test was used to analyze the qualitative data between the two groups.

Ethical consideration

The Ethics Committee at Prince Sultan Military Medical City granted the necessary approval for the study. The objective of the study was clearly communicated to all participants, and only those who provided informed consent were included in the research. Participants were assured that their confidentiality would be maintained, and the questionnaire did not seek any personal information.

## Results

A total of 417 participants were enrolled in the study. The mean age of the participants was 20 ± 2.03 years, the mean weight was 69.3 ± 18.8 kg, the average height was 167.8 ± 11.5 cm, and the mean body mass index (BMI) was 24.2 ± 5.3. Of the participants, 227 (54.4%) were male, and 190 (45.6%) were female. Regarding academic year, 110 (26.4%) were in the third year, 98 (23.5%) were in the first year, 86 (20.6%) were in the second year, 68 (16.3%) were in the fifth year, and 55 (13.2%) were in the fourth year. In terms of marital status, 409 (98.1%) were single, and 8 (1.9%) were married (Table 1).

**Table 1:** Characteristics of the study participants (n=417)

Variable	Mean ± SD
Age (years)	20.8 ± 2.03
Weight (Kg)	69.3 ± 18.8
Height (cm)	167.8 ± 11.5
BMI (Kg/m2)	24.2 ± 5.3
Gender	N (%)
Male	227 (54.4)
Female	190 (45.6)
Academic year	
1st year	98 (23.5)
2nd year	86 (20.6)
3rd year	110 (26.4)
4th year	55 (13.2)
5th year	68 (16.3)
Marital status	
Single	409 (98.1)
Married	8 (1.9)

**Figure 1:**
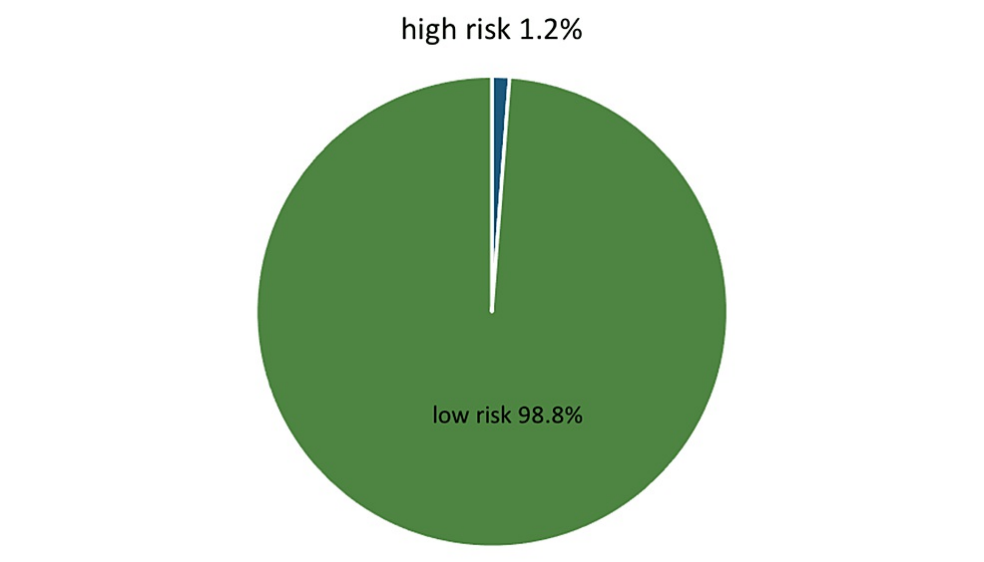
Risk of diabetes mellitus type 2 among medical students

Out of a total of 417 participants, the mean T2DM risk score was found to be 1.83 ± 1.32 out of 11. Furthermore, 412 (98.8%) of the participants were found to have a low-risk score for T2DM, while only five (1.2%) of the participants were found to had a higher risk of developing T2DM (Figure 1).

Approximately 321 (77%) of the participants reported that they had checked their weight and calculated their BMI, with four (80%) of those at higher risk for T2DM reporting having done so within the past year, while the remaining 96 (23%) had not checked. However, no statistically significant association was found between checking weight and BMI and T2DM risk (p=1.000). Out of the total 417 participants, 157 (37.6%) had checked their blood sugar level in the past year. Among those with a high risk of DM, two out of five (40%) had checked their blood sugar level in the past year, while 260 (62.4%) of the participants had not checked their blood sugar level in the past year. Among those with a high risk of DM, three out of five (60%) had not checked their blood sugar level in the past year.

When asked about which gender is at higher risk of developing T2DM, about 150 (36%) of the participants mentioned that females are at increased risk of developing T2DM, whereas 108 (25.9%) of the participants stated that males have a higher risk of developing T2DM than females, and 159 (38.1%) reported that the risk is equal, with no difference between males and females regarding the risk of developing T2DM. The p-value for this analysis was 0.311. Additionally, 389 (93.3%) of the participants believed that the risk of developing T2DM increases with age, while two (0.5%) thought that the risk decreases with age, and 26 (6.2%) believed that the risk is equal regardless of age. Obesity was reported as a risk factor for T2DM by 409 (98.1%) of the participants, while smoking was mentioned by 241 (57.8%) as a risk factor. However, no significant differences were found between participants' awareness about risk factors and their actual risk of developing T2DM (p=1.000). Family history of DM was identified as a risk factor by 402 (96.4%) participants, and a sedentary lifestyle was mentioned by 373 (89.4%) participants as a risk factor for T2DM. However, no significant association was found between awareness of family history of DM and a sedentary lifestyle with the risk of developing T2DM (p-value 1.000 and 0.660, respectively). Hyperthyroidism was reported as a risk factor for T2DM by about 150 (36%) of the participants. History of gestational DM was mentioned by 337 (80.8%) of the participants, of which five were considered to be at a higher risk of developing T2DM. Hypertension was reported as a risk factor for T2DM by 224 (53.7%) of the participants, and three of them, which constitutes about 60% of the participants with a high risk of developing T2DM, also reported having hypertension. Awareness about hypertension as a risk factor for DM was not found to be significantly associated with the risk of DM. Intravenous drug abuse was reported by 100 (24%) of the participants as a risk factor for T2DM, whereas 317 (76%) did not think that intravenous drug abuse is a risk factor for T2DM. Knowledge and awareness about intravenous drug abuse and DM were not found to be significantly associated with the risk of developing T2DM (p=1.000).

**Table 2:** T2DM risk factor awareness and its association with having low or high risk of T2DM T2DM - type 2 diabetes mellitus

Variable	N (%)	DM risk		p-value
		Low	High	
During the past year, have you checked your weight and calculated your BMI?				
Yes	321 (77)	317 (76.9)	4 (80)	1
No	96 (23)	95 (23.1)	1 (20)	
Have you checked your blood sugar level in the past year?				
Yes	157 (37.6)	155(37.6)	2 (40)	1
No	260 (62.4)	257 (62.4)	3 (60)	
Who among the genders has a higher risk for diabetes mellitus type 2?				
Male	108 (25.9)	106 (25.7)	2 (40)	0.311
Female	150 (36)	150 (36.4)	0 (0)	
Equal risk	159 (38.1)	156 (37.9)	3 (60)	
What do you know about the association between DM risk and age?				
Increases with age	389 (93.3)	384 (93.2)	5 (100)	1
Decreases with age	2 (0.5)	2 (0.5)	0 (0)	
Equal risk regardless of age	26 (6.2)	26 (6.3)	0 (0)	
What are the factors associated with higher risk of DM type 2?				
Obesity				
Yes	409 (98.1)	404 (98.1)	5 (100)	1
No	8 (1.9)	8 (1.9)	0 (0)	
Smoking				
Yes	241 (57.8)	236 (57.3)	5 (100)	0.076
No	176 (42.2)	176 (42.7)	0 (0)	
Family history of DM				
Yes	402 (96.4)	397 (96.4)	5 (100)	1
No	15 (3.6)	15 (3.6)	0 (0)	
Sedentary lifestyle				
Yes	373 (89.4)	368 (89.3)	5 (100)	0.66
No	44 (10.6)	44 (10.7)	0 (0)	
Hyperthyroidism				
Yes	150 (36)	148 (35.9)	2 (40)	1
No	267 (64)	264 (64.1)	3 (60)	
History of gestational DM during pregnancy				
Yes	337 (80.8)	332 (80.6)	5 (100)	0.588
No	80 (19.2)	80 (19.4)	0 (0)	
Hypertension				
Yes	224 (53.7)	221 (53.6)	3 (60)	1
No	193 (46.3)	191 (46.4)	2 (40)	
Intravenous drug abuse				
Yes	100 (24)	99 (24)	1 (20)	1
No	317 (76)	313 (76)	4 (80)	

## Discussion

This study aimed to evaluate the awareness of medical students in Riyadh, Saudi Arabia, about the risk factors associated with T2DM. Assessing the level of knowledge about these risk factors is crucial in understanding the factors that contribute to the development of the disease, including lifestyle behaviors that may increase or decrease the risk of T2DM [11].

The average age of the participants was found to be 20 years old, with a mean BMI of 24.2. Approximately half (54.4%) of the participants were males. The mean DM risk score, out of a total of 11, was found to be 1.83. The vast majority (98.8%) of the participants were found to have a low-risk score for T2DM, while only 1.2% were found to have a higher risk of developing T2DM. This percentage was significantly lower than that reported in a parallel study conducted by Tomic et al., in which a 16.3% risk was noted [12]. More than two-thirds (77%) of the participants reported having checked their weight and calculated their BMI, and among those with a high risk of DM, 80% reported having done so during the last year. Body mass index as a risk factor for T2DM was demonstrated in the parallel study carried out by Ganz et al. [13]. More than one-third (37.6%) of the participants reported having checked their blood sugar levels in the past year, and two of them (40%) of those with high risk had checked their blood sugar level in the last year. Our study revealed no significant difference between males and females regarding the risk of developing T2DM.

Slightly more than one-third (36%) of the participants mentioned that females are at increased risk of developing T2DM, which is contradictory to the findings reported in the study conducted by Oraii et al., which showed that males are at increased risk of developing T2DM [14]. The vast majority (93.3%) of the participants reported that the risk of DM increases with age. Concerning modifiable factors associated with a higher risk of T2DM, obesity was mentioned as a risk factor by the vast majority (98.1%) of the participants, while smoking was reported by more than half (57.8%) as a risk factor for T2DM. The vast majority (96.4%) of the participants mentioned a family history of DM as a risk factor. A sedentary lifestyle was also mentioned by the majority (89.4%) of the participants, and this was found to be consistent with the findings reported in the study by Gyawali, which reported an increased risk of T2DM for the aforementioned factors [15]. Hyperthyroidism was reported as a risk factor for T2DM by more than one-third (36%) of the participants. The history of gestational DM was mentioned by the vast majority (80.8%) of the participants, and five of them were considered to have a higher risk of developing T2DM. The McIntyre study also noted findings similar to these findings, which revealed gestational DM as a risk factor for T2DM [16]. Intravenous drug abuse was reported by one-quarter (24%) of the participants as a risk factor for T2DM, whereas more than two-thirds (76%) do not think that intravenous drug abuse is a risk factor for T2DM. Knowledge and awareness about intravenous drug abuse and DM were not found to be significantly associated with the risk of developing T2DM. Hypertension was reported as a risk factor for T2DM by over half (53.7%) of the participants. Out of the total participants, five individuals were identified as being at high risk for developing T2DM. Among these five participants, three individuals, constituting 60% of the high-risk group, reported hypertension as a risk factor for T2DM. This was found to be consistent with the findings of a Korean study, which revealed that hypertension is an independent risk factor for developing T2DM [17].

Our research is subject to certain limitations. Firstly, the characteristics of the sample were limited to a single university in Saudi Arabia, which may limit the generalizability of our findings to other settings. Secondly, the cross-sectional design of our study makes it impossible to establish a causal relationship between the variables. Lastly, selection bias may have been a potential limitation in our study.

## Conclusions

The study revealed that the majority of participants exhibited a high level of knowledge and awareness concerning T2DM. Merely 1.2% of the participants were identified as being at an elevated risk of developing T2DM. Notably, there was no significant correlation observed between having a high- or low-risk score for T2DM and possessing a high or low awareness level of the disease. To enhance knowledge and awareness levels, it is suggested to promote health education programs, including community events focused on T2DM. Additionally, leveraging the influential role of media as a powerful tool for knowledge dissemination could contribute to a significant response in increasing awareness. Improving these areas holds promise for addressing T2DM effectively.
